# Warming-induced tipping points of Arctic and alpine shrub recruitment

**DOI:** 10.1073/pnas.2118120119

**Published:** 2022-02-22

**Authors:** Xiaoming Lu, Eryuan Liang, Flurin Babst, J. Julio Camarero, Ulf Büntgen

**Affiliations:** ^a^State Key Laboratory of Tibetan Plateau Earth System, Resources and Environment, Institute of Tibetan Plateau Research, Chinese Academy of Sciences, 100101 Beijing, China;; ^b^School of Natural Resources and the Environment, University of Arizona, Tucson, AZ 85721;; ^c^Laboratory of Tree-Ring Research, University of Arizona, Tucson, AZ 85721;; ^d^Instituto Pirenaico de Ecología, Consejo Superior de Investigaciones Científicas (IPE-CSIC), 50059 Zaragoza, Spain;; ^e^Department of Geography, University of Cambridge, Cambridge CB2 3EN, United Kingdom;; ^f^Swiss Federal Research Institute, 8903 Birmensdorf, Switzerland;; ^g^Global Change Research Institute, Czech Academy of Sciences (CzechGlobe), 603 00 Brno, Czech Republic;; ^h^Department of Geography, Faculty of Science, Masaryk University, 613 00 Brno, Czech Republic

**Keywords:** alpine, Arctic, climate change, shrub recruitment, tipping point

## Abstract

Shrub recruitment, a key component of vegetation dynamics beyond forests, is a highly sensitive indicator of climate and environmental change. Warming-induced tipping points in Arctic and alpine treeless ecosystems are, however, little understood. Here, we compare two long-term recruitment datasets of 2,770 shrubs from coastal East Greenland and from the Tibetan Plateau against atmospheric circulation patterns between 1871 and 2010 Common Era. Increasing rates of shrub recruitment since 1871 reached critical tipping points in the 1930s and 1960s on the Tibetan Plateau and in East Greenland, respectively. A recent decline in shrub recruitment in both datasets was likely related to warmer and drier climates, with a stronger May to July El Niño Southern Oscillation over the Tibetan Plateau and a stronger June to July Atlantic Multidecadal Oscillation over Greenland. Exceeding the thermal optimum of shrub recruitment, the recent warming trend may cause soil moisture deficit. Our findings suggest that changes in atmospheric circulation explain regional climate dynamics and associated response patterns in Arctic and alpine shrub communities, knowledge that should be considered to protect vulnerable high-elevation and high-latitude ecosystems from the cascading effects of anthropogenic warming.

In hosting the world’s coldest distribution limits of shrubs, the Arctic and the Tibetan Plateau share some similar ecosystems (1, 2). In addition, their climate is strongly influenced by large-scale atmospheric circulations, such as those captured by the Arctic Oscillation (AO), Atlantic Multidecadal Oscillation (AMO), and El Niño Southern Oscillation (ENSO) (3–5). As shrub vegetation depends on climate, it is highly responsive to changes in atmospheric circulation (6). During the 20th century, shrub encroachment into Arctic and alpine grasslands has contributed to changes in carbon storage, biodiversity, and feedbacks to regional climate (7). Nevertheless, the long-term interplay between shrub expansion and atmospheric circulation in these cold biomes has remained poorly characterized and quantified.

The temporal dynamics of shrub expansion can be indirectly explored by assessing shrub recruitment, which is one of the most sensitive indicators of climate warming (7). Despite the accelerated warming and greening trends in high latitudes and altitudes, recent reductions in shrub recruitment have been observed in coastal East Greenland and on the Tibetan Plateau (8, 9). It appears possible that these trends have been caused by climate change-induced shifts in hydrothermal conditions beyond the regional optimum for major shrub species. Here, we hypothesize that changes in atmospheric circulation patterns may significantly affect shrub recruitment in Greenland and on the Tibetan Plateau.

## Results

**Fig. 1.**
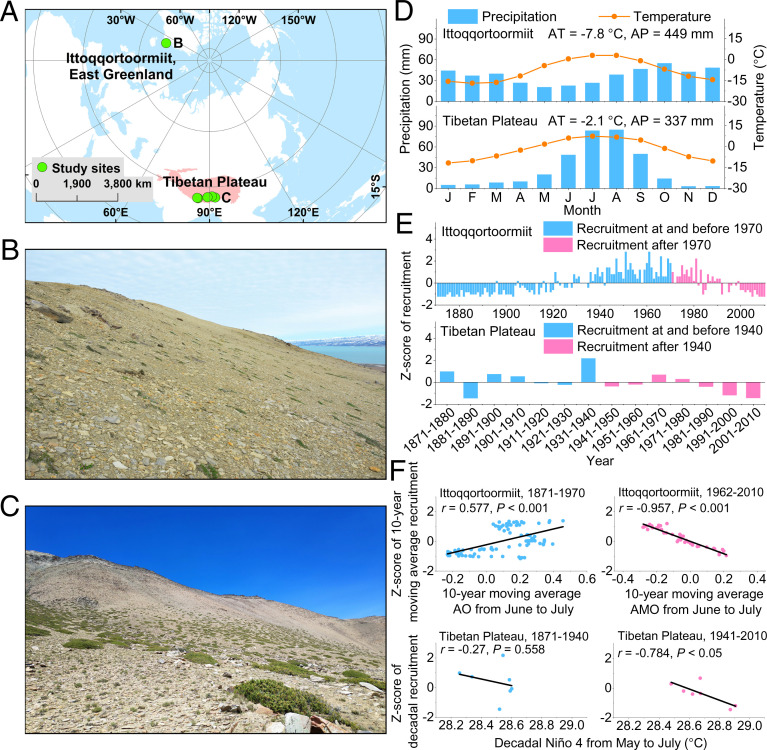
Study sites, temporal courses of shrub recruitment, and their relationships with atmospheric circulation patterns. The study sites (*A*) and views of study shrublands in Greenland arctic tundra (*B*, *Rhododendron lapponicum* and other species; photo taken by U.B.), and on the Tibetan Plateau (*C*, *Juniperus pingii* var. *wilsonii*). Image credit: X.L. (*D*) Monthly climate in both regions. Annual mean temperature (AT) and total precipitation (AP) are also shown. (*E*) Annual shrub recruitment data in Greenland and decadal shrub recruitment on the Tibetan Plateau. (*F*) Linear relationships calculated between the atmospheric circulation indices and shrub recruitment.

Recruitment of high Arctic shrub species peaked in about 1961 to 1970 in coastal east Greenland near Ittoqqortoormiit (Fig. 1 *A*–*E*), whereas the recruitment peak of alpine juniper shrub occurred three decades earlier on the Tibetan Plateau (1931 to 1940). Shrub recruitment declined subsequently in both study regions (Fig. 1*E* and *SI Appendix*).

**Fig. 2.**
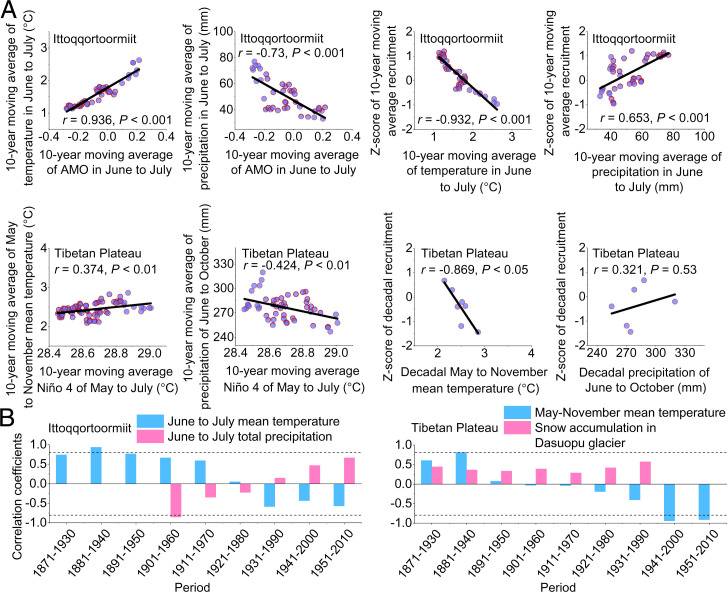
Linear regressions between shrub recruitment series and climate, and 60-y moving window Pearson correlations. (*A*) Linear regressions calculated between the atmospheric circulation indices and local climate variables during the periods with recruitment decline. Linear regressions were calculated between the local climate variables and shrub recruitment in each study region. The analysis period is 1962 to 2010 for Greenland. For the Tibetan Plateau, the time spans of temperature and precipitation data used for the analyses are 1932 to 2010 and 1951 to 2010, respectively. (*B*) Moving window Pearson correlations calculated between the decadal recruitment series and temperature, and precipitation and snow accumulation data in Greenland and the Tibetan Plateau sites. The horizontal dashed lines indicate the significance level at *P* = 0.05.

Shrub recruitment was highly correlated with atmospheric circulation indices (Fig. 1*F*). Specifically, shrub recruitment in Greenland was strongly related to variability of the AO and AMO. AO from June to July was significantly and positively related with recruitment from 1871 to 1970, whereas during recent decades (1962 to 2010), the AMO from June to July showed a significant negative correlation with recruitment (*r* = 0.395 in 1871 to 1970; *r* = −0.934 in 1962 to 2010; *P* < 0.001 in both cases and considering detrended series). June to July AMO drove the regional mean temperature of those months, with warmer conditions being associated with a higher AMO index (Fig. 2*A* and Dataset S1). However, decreased precipitation was also associated with high AMO values. Consequently, temperature and precipitation had negative and positive effects on shrub recruitment, respectively. May to July Niño 4 had negative associations with shrub recruitment on the Tibetan Plateau (Fig. 1*F*) (*r* = −0.808 in 1871 to 1940, *P* < 0.05; *r* = −0.73 in 1941 to 2010, *P* = 0.06 for detrended series). Increased mean temperature in May to November and lower June to October precipitation were also related to Niño 4 since the 1930s (Fig. 2*A* and Dataset S1). In addition, recruitment was significantly and negatively correlated with decadal mean May to November temperature in 1941 to 2010. A positive correlation was found between recruitment and monsoon season precipitation.

Moving correlation analyses showed that the sign of the temperature sensitivity of recruitment shifted from positive to negative after passing recruitment tipping points in both locations (Fig. 2*B*).

## Discussion

There is growing concern that Arctic and alpine ecosystems may reach tipping points under accelerating climate change (10–12). Our study now adds strong evidence that climate-induced tipping points during the past decades have already reversed the formerly increasing trend in shrub recruitment across these cold biomes. Moreover, our results importantly provide a long-term context for the quantification of shrub recruitment variations in relation to changes in climatic circulation patterns.

The observed variability in shrub recruitment and the underlying relationships with changes in atmospheric circulation patterns indicate complex responses of spatial and temporal shrub regeneration and expansion in the Arctic and the Tibetan Plateau. For example, shrub recruitment peaked in different time periods (Fig. 1*E*), suggesting a high degree of heterogeneity of shrub encroachment in these distant treeless areas. We hypothesize that the accelerated greening trends prior to peak recruitment were associated with climate warming in both arctic and alpine regions (13).

The declines of shrub recruitment likely resulted from drought stress associated with changes in large-scale atmospheric circulation patterns. In Greenland and on the Tibetan Plateau, high AMO and Niño 4 were associated with a warmer and drier climate (Fig. 2*A* and Dataset S1), leading to unfavorable recruitment conditions with low soil moisture. Despite the long-standing notion that warming has accelerated greening across those cold regions, some studies have demonstrated that tundra vegetation exhibited strong, locally contingent responses to climate (14). In particular, shrub recruitment is strongly limited by the available soil moisture (14). Future warming may thus further impair shrub recruitment or increase mortality rates by intensifying soil drying. Similar to our results, shrub growth at more than one-third of the Pan-Arctic sites has already indicated warming-induced drought stress, showing an early warning signal of a state shift in shrub communities (15).

This study highlights the tight links between large-scale atmospheric circulations and shrub recruitment in the Arctic and on the Tibetan Plateau. Atmospheric circulation patterns could thus be used to forecast spatiotemporal shifts in shrub recruitment, and thereby assist in projecting future vegetation shifts in cold biomes. Our data also indicated that the optimal climate for shrub recruitment has already been passed in two remote and ecologically important cold regions.

## Materials and Methods

Shrub recruitment series were established from two datasets, including 2,770 individuals in Ittoqqortoormiit and on the Tibetan Plateau (8, 9). The climate data mainly used were obtained from the Climate Explorer (http://climexp.knmi.nl/) and National Oceanic and Atmospheric Administration websites (https://psl.noaa.gov/gcos_wgsp/Timeseries/). Linear regressions and moving Pearson correlation analyses were applied to assess the relationships between shrub recruitment series and climate. See *SI Appendix* for further details.

## Supplementary Material

Supplementary File

Supplementary File

## Data Availability

All shrub recruitment data used for analyses are available in Dataset S1. All other study data are included in the main text and supporting information.
