# Supplementary material for: Warming-induced tipping points of Arctic and alpine shrub recruitment
Source: Proc Natl Acad Sci U S A. 2022 Feb 22;119(9):e2118120119. doi: 10.1073/pnas.2118120119 (PMC8917345; doi:10.1073/pnas.2118120119)
Supplement: Supplementary File [file pnas.2118120119.sapp.pdf]

PNAS
www.pnas.org

Supplementary Information for

Warming-induced tipping points of Arctic and alpine shrub recruitment

Xiaoming Lu[a], Eryuan Liang[a,*], Flurin Babst[b,c], J. Julio Camarero[d], Ulf Büntgen[e,f,g,h]

[a]State Key Laboratory of Tibetan Plateau Earth System, Resources and Environment (TPESRE), Institute of Tibetan Plateau Research, Chinese Academy of Sciences, Beijing 100101, China
[b]School of Natural Resources and the Environment, University of Arizona, Tucson, Arizona, USA
[c]Laboratory of Tree-Ring Research, University of Arizona, Tucson, Arizona, USA
[d]Instituto Pirenaico de Ecología (IPE-CSIC), Zaragoza, Spain
[e]Department of Geography, University of Cambridge, Cambridge CB2 3EN, UK
[f]Swiss Federal Research Institute (WSL), 8903 Birmensdorf, Switzerland
[g]Global Change Research Institute of the Czech Academy of Sciences (CzechGlobe), 603 00 Brno, Czech Republic
[h]Department of Geography, Faculty of Science, Masaryk University, 613 00 Brno, Czech Republic

[*]Corresponding Author: Eryuan Liang; Email: liangey@itpcas.ac.cn

This PDF file includes:
Extended Methods
SI References

Supplementary Information Text

Extended Methods

Study area and climate conditions

The Ittoqqortoormiit (70.43° N, 21.99° W) study site is located in eastern Greenland (1) (elevation from 1 to 320 m a.s.l., Fig. 1). Our four alpine shrubline sites (29.40°–30.53° N, 84.87°–92.36° E, 4933–5220 m a.s.l.) are situated on the southern Tibetan Plateau (2).

The Ittoqqortoormiit site is characterized by a tundra climate and has a short and warm growing season (June to August) and a mean annual temperature of −7.8 °C, with dark winter conditions lasting for more than seven months (Fig. 1). According to the gridded Climate Research Unit dataset (3) (CRU TS 4.04, http://climexp.knmi.nl/), annual precipitation for the Ittoqqortoormiit site during the period of 1950–2010 was 449 mm, with precipitation mainly falling from October to March. Our study sites on the southern Tibetan Plateau lie in an alpine semi-arid climate zone with an annual mean temperature of −2.1 °C (1950–2010, CRU data) and a total precipitation of 337 mm per year (4) (1951–2010).

Shrub recruitment data

To examine long-term changes in shrub recruitment across tundra and alpine regions, we revisited two published datasets of shrub recruitment that contain a total of 2,770 recorded individuals (1, 2). The Ittoqqortoormiit dataset included 10 shrub species and 871 individuals (1). The annually resolved recruitment chronology developed at this site covered the period of 1807–2010. On the south-central Tibetan Plateau, we developed a decadal juniper shrub

(*Juniperus pingii* var. *wilsonii*) recruitment chronology by averaging the recruitment series of four sites at the world's highest shrublines since 1601 (2). All recruitment series were then transformed into z-scores to facilitate comparisons of recruitment variability between sites. Lastly, shrub recruitment data were truncated in 1871, because the time coverage of available atmospheric circulation pattern records only dates back that far.

Climate data

The Arctic Oscillation (AO) is a natural climatic circulation pattern in high latitudes that emerges from sea level pressure anomalies at the 1000-hPa height (5). Another important atmospheric circulation affecting the Arctic is the Atlantic Multidecadal Oscillation (AMO, 6), defined as a coherent mode of natural sea surface temperature variability of the North Atlantic Ocean with a 60–80 year cycle (7). The El Niño Southern Oscillation (ENSO) originates in the tropical Pacific Ocean due to changes in sea surface temperatures. It is the largest external climatic forcing with multiple pathways of influencing the global climate (8). Specifically, there is an inverse relationship between ENSO and the Indian summer monsoon, which affects the growing season rainfall on the Tibetan Plateau (9). We used the monthly climate time-series of AO from the 20th century reanalysis (10), unsmoothed AMO and El Niño 4 (averaged sea surface temperature from the region of 5° S–5° N and 160° E–150° W, series available at https://psl.noaa.gov/gcos_wgsp/Timeseries/). The time span of these records ranges from 1871 to 2010.

Monthly CRU data of mean temperatures and total precipitation were obtained for the Ittoqqortoormiit site (0.5° resolution, period 1901–2010). We also obtained monthly CRU

mean temperatures and APHRODITE 0.25°-gridded total precipitation (4) (period 1951–2010) of the four study sites on the Tibetan Plateau. The average or summed series of temperature and precipitation data, respectively, were then used for analyses. Finally, we extracted long-term 5°-gridded temperature (11) (HadCRUT4, period 1850–2020) for Ittoqqortoormiit and the Tibetan Plateau and snow accumulation data in the Tibetan Plateau Dasuopu glacier (12) (i.e., a precipitation proxy) for our analyses.

Data analyses

Shrub recruitment may lag behind climate variability by several years (1), as shrubs need considerable time for seed maturation and seedling establishment. In this study, climate data (AO, AMO and gridded CRU data) and z-scores of annually shrub recruitment at the Ittoqqortoormiit site were, therefore, transformed into lower frequency (10-year moving averages) time series to reduce the impact of lagged climate-recruitment effects. For the Tibetan Plateau, the climate data were first transformed into 10-year moving average series to investigate the relationships between the ENSO and gridded temperature and precipitation. Moreover, decadal gridded climate data and El Niño 4 were computed to detect their relationships with recruitment dynamics. For both study regions, we also calculated decadal time series of recruitment, climate series including mean temperature, precipitation, and snow accumulation data in Dasuopu for moving window Pearson correlation analyses. Each recruitment series was divided into two time series according to their peaks (tipping points). Linear regressions were then applied to explore the relationships between large-spatial climate circulations and recruitment series by using the above 10-year moving average or

decadal resolved series. To test whether such relationships persist, we examined the relationships between the detrended recruitments series and climate circulations. We removed the year-related trend from those recruitment and climate series by extracting or dividing by the values calculated from linear regressions with the year as the only independent variable. Recruitment series were also divided into two segments for the detrending process. To detect how atmospheric circulation patterns related to shrub recruitment declines, linear regressions were firstly used to evaluate the impacts of atmospheric circulations on the regional climate at the study sites. We then repeated the same approach to analyze the relationships between regional climate variability and recruitment. A 60-year moving window Pearson correlation analysis was used to detect whether the relationships between recruitment and its main limiting climate factors changed after reaching tipping points at each site.

SI References

1. U. Büntgen *et al.*, Temperature-induced recruitment pulses of Arctic dwarf shrub communities. *J. Ecol.* **103**, 489–501 (2015).
2. X. Lu *et al.*, Past the climate optimum: Recruitment is declining at the world's highest juniper shrublines on the Tibetan Plateau. *Ecology* **100**, e02557 (2019).
3. I. Harris, P. D. Jones, T. J. Osborn, D. H. Lister, Updated high-resolution grids of monthly climatic observations-the CRU TS3.10 Dataset. *Int. J. Climatol.* **34**, 623–642 (2014).
4. A. Yatagai *et al.*, APHRODITE Constructing a long-term daily gridded precipitation dataset for Asia based on a dense network of rain gauges. *B. Am. Meteorol. Soc.* **93**,

1401–1415 (2012).

5. D. W. J. Thompson, J. M. Wallace, The Arctic Oscillation signature in the wintertime geopotential height and temperature fields. *Geophys. Res. Lett.* **25**, 1297–1300 (1998).
6. P. Chylek, C. K. Folland, G. Lesins, M. K. Dubey, M. Wang, Arctic air temperature change amplification and the Atlantic Multidecadal Oscillation. *Geophys. Res. Lett.* **36**, L14801 (2009).
7. D. B. Enfield, A. M. Mestas-Nuñez, P. J. Trimble, The Atlantic Multidecadal Oscillation and its relation to rainfall and river flows in the continental US. *Geophys. Res. Lett.* **28**, 2077–2080 (2001).
8. N. A. Rayner *et al.*, Global analyses of sea surface temperature, sea ice, and night marine air temperature since the late nineteenth century. *J. Geophys. Res-Atmos.* **108**, 4407 (2003).
9. E. C. Gill, B. Rajagopalan, P. Molnar, Subseasonal variations in spatial signatures of ENSO on the Indian summer monsoon from 1901 to 2009. *J. Geophys. Res-Atmos.* **120**, 8165–8185 (2015).
10. G. P. Compo *et al.*, The twentieth century reanalysis project. *Q. J. Roy. Meteor. Soc.* **137**, 1–28 (2011).
11. C. P. Morice, J. J. Kennedy, N. A. Rayner, P. D. Jones, Quantifying uncertainties in global and regional temperature change using an ensemble of observational estimates: The HadCRUT4 data set. *J. Geophys. Res-Atmos.* **117**, D08101 (2012).
12. L. G. Thompson *et al.*, A high-resolution millennial record of the south Asian monsoon from Himalayan ice cores. *Science* **289**, 1916–1919 (2000).
